# Ectomycorrhiza resilience and recovery to extreme flood events in *Tuber aestivum* and *Quercus robur*

**DOI:** 10.1007/s00572-021-01035-4

**Published:** 2021-05-13

**Authors:** P. W. Thomas

**Affiliations:** 1 Faculty of Natural Sciences, University of Stirling, Stirling, FK9 4LA UK; 2 Mycorrhizal Systems Ltd, Lancashire, PR25 2SD UK

**Keywords:** Flooding, Climate change, Soil saturation, *Quercus robur* (oak), *Tuber aestivum* (summer truffle), Mycorrhiza

## Abstract

Very little is known about the impact of flooding and ground saturation on ectomycorrhizal fungi (EcM) and increasing flood events are expected with predicted climate change. To explore this, seedlings inoculated with the EcM species *Tuber aestivum* were exposed to a range of flood durations. Oak seedlings inoculated with *T. aestivum* were submerged for between 7 and 65 days. After a minimum of 114-day recovery, seedling growth measurements were recorded, and root systems were destructively sampled to measure the number of existing mycorrhizae in different zones. Number of mycorrhizae did not display correlation with seedling growth measurements. Seven days of submersion resulted in a significant reduction in mycorrhizae numbers and numbers reduced most drastically in the upper zones. Increases in duration of submersion further impacted mycorrhizae numbers in the lowest soil zone only. *T. aestivum* mycorrhizae can survive flood durations of at least 65 days. After flooding, mycorrhizae occur in higher numbers in the lowest soil zone, suggesting a mix of resilience and recovery. The results will aid in furthering our understanding of EcM but also may aid in conservation initiatives as well as providing insight for those whose livelihoods revolve around the collection of EcM fruiting bodies or cropping of the plant partners.

**Supplementary information:**

The online version contains supplementary material available at 10.1007/s00572-021-01035-4.

## Introduction

The term mycorrhiza describes a symbiotic relationship between plants and fungi, in which the plant partner provides photosynthetically derived carbon in exchange for nutrients (Smith and Read 2010). At least 86% of angiosperms form mycorrhiza and arbuscular mycorrhiza (AM), ectomycorrhiza (EcM) and ericoid mycorrhiza (ErM) are geographically the most widespread (Brundrett 2009). AM species are the most abundant and these appear relatively tolerant to hostile conditions such as high levels of soil salinity, occurring in wetland environments from calcareous fen to saltmarsh and mangroves (Rozema et al. 1986; Zhouying et al. 2016). However, AM are not ubiquitous in waterlogged environments (Radhika and Rodrigues 2007) and the degree of colonisation may decrease with increased flooding along wetland gradients (Wang et al. 2010). The occurrence of some AM species within waterlogged environments has been suggested, in some cases, to be driven by the presence of well-developed aerenchyma (air chambers) within the root system that may reduce anaerobiosis stress, such as low oxygen tension and the accumulation of reduced toxic substances (Rozema et al. 1986; Wang et al. 2010). These chambers benefit the plant but also its mycorrhizal endophyte. Aerenchyma tissue, through the facilitation of gas exchange from above ground plant parts to the rhizosphere, may influence the survival of mycorrhizal fungi and other aerobic microorganisms (Cooke and Lefor 1998).

In contrast to AM, the nature of EcM with an intercellular interface presents a greater structural vulnerability to saturated soil conditions. Indeed, it has been hypothesised that low EcM levels in some field studies may be an indication of regularly flooded soils (Katanić et al. 2015). However, the mycelia produced by EcM fungi can be categorised as either hydrophobic or hydrophilic and species within the latter category may be better able to resist saturated soil conditions. Hydrophobicity may be an adaptation to retain water in dry soil and prevent inundation in wet soil (Unestam and Sun 1995; Barnes et al. 2018). Although the impact of drought and water scarcity on microbial communities, including EcM, has been fairly well studied, there are comparatively few reports that focus on flooding and soil saturation (Johnson 2018). However, there are some intriguing insights. For example, *Alnus glutinosa* (L.) Gaertn. is a tree species that occurs in areas with permanent high ground water levels and frequent inundation. Despite this, *A. glutinosa* may associate with up to 40 different EcM species although in one study, it was observed that dryer soils yielded nearly 3× the unique EcM species of wetter soils (Tedersoo et al. 2009). *A. glutinosa* may also form AM associations and this decline in EcM richness in wetter conditions may be symptomatic of AM dominance in such circumstances, although this has not been experimentally verified.

Differing responses of hydrophobic or hydrophilic EcM has also been verified experimentally. For example, in a study with the tree species *Pinus sylvestris*, the EcM species *Suillus bovinus* proved particularly vulnerable to high water levels, with colonisation of the root system unable to occur when the system was flooded for just 2 min a day, four times a week. By contrast, *Thelephora terrestris*, *Laccaria laccata* and *Hebeloma crustuliniforme* were resilient to such conditions (Stenström 1991). General EcM colonisation has been shown to be inhibited at high soil moisture levels (Lodge 1989) and declines in EcM in response to waterlogged conditions have also been observed outside the laboratory. Field observations of a *Salix viminalis* L. plantation in England (UK) present declines in both the abundance and species richness of EcM communities in response to increased ground saturation caused by localised extreme weather events. Interestingly, these changes were observed without any alteration in major soil physio‐chemical properties driven by potentially anoxic soil conditions, having been recorded. Further, when the results were subdivided, it was observed that this decline did not occur in hydrophilic EcM (Barnes et al. 2018). Although such studies allow us to draw conclusions such that EcM in general show sensitivity to flooding, with significant interspecies variation, there is much detail we still do not know. For example, how long can established EcM survive waterlogged conditions and what is the resilience and recovery within the system? The very basic question of how long EcM can tolerate saturated conditions has not previously been addressed.

Flooding and extreme precipitation events have recently been increasing and anthropogenically driven climate change is thought to be a driving force. Globally, the number of floods and other hydrological events have increased fourfold since 1980 and have doubled since 2004 (Hov et al. 2018). Projected atmospheric warming is forecast to increase future flood risk at a global scale. For example, with a global warming of 4 °C, countries representing more than 70% of the global population are projected to face increases in flood risk in excess of 500% (Alfieri et al. 2017). In the face of such dramatic change, investigating how EcM respond to such events may seem trivial but understanding such responses are important for a number of reasons. Firstly, a deeper knowledge will allow us to better understand the impact of such events on ecosystems at a range of spatiotemporal scales and can help inform conservation initiatives. Secondly, a range of food crops such as the majority of temperate nut production as well as commercial timber activities are dominated by species that form EcM. By better understanding the resilience and tolerances of EcM, we can better protect these industries. Thirdly, the local collection of fruit bodies from a number of EcM fungi may be quite significant from a socioeconomic point of view (e.g. Saito and Mitsumata 2008) as well as forming an important component of local cultural identity (Samils et al. 2008). Economically, the most important edible mycorrhizal fungi are from the genus *Tuber*, the hypogeal fruiting bodies of which are known collectively as truffles. In a recent analysis of non-wood forest products of Europe, the economic value of harvested truffles was estimated at ~ 3.1 billion € yr^−1^ (Lovrić et al. 2020). Truffles are widely cultivated, we know they face an uncertain future due to predicted climate change (Thomas and Büntgen 2019) and may be sensitive to soil moisture (Olivera et al. 2014) but a more nuanced understanding of the biology may allow us to better prepare.

In order to better understand the impact of flooding on EcM, tree saplings inoculated with *Tuber aestivum* (the summer truffle) were subjected to submersion for a range of time periods from 7 to 65 days. The impact on EcM was assessed after a recovery period. *T. aestivum* was chosen so that the results could also help inform truffle cultivators and managers of naturally producing truffle woods, to prepare and respond to flood events.

## Materials and methods

### Preparation of inoculated seedlings

To prepare the inoculated saplings, acorns from mature *Quercus robur* trees in UK seed region 403 (see: Herbert et al. 1999) were collected in autumn 2014. Seed was cleaned for 5 min with 5% sodium hypochlorite and then thoroughly rinsed to remove all residue. The seed was sown in spring 2015 into a potting mix of 70% sandy loam and 30% perlite. The mix was heat treated and the pH was adjusted to 7.6 using ground lime. Individual plants were inoculated with a sporal solution of macerated *T. aestivum* sporocarps of English provenance, with no site selection criteria of inoculum source applied. Each plant received a dose equivalent to 1 g of fresh sporocarp, applied directly and whilst in the growing medium. The plants were grown-on for a full season, under glass in Lancashire (UK) in 17 cm deep Rootrainer pots with a volume of 350 cc (Tildenet Group Ltd, UK). In October 2015, a subset of plants was destructively sampled to confirm colonisation with *T. aestivum* using the protocol outlined under *Analysis Methodology*. Seed that had not formed a shoot by this time point were eliminated from the experiment, as were any seedling under 5 cm.

### Submersion treatment

On the 25^th^ of January 2016, all plants were removed from the glasshouse and treatment groups were suspended in an immersion tank holding rainwater, in the open air. On the same day, 10 plants for a control group were placed on a growing table adjacent to the immersion tank. The water level of the immersion tank was maintained at approximately 1 cm above the soil line of the pots. No water exchange was carried out, nor intentional mixing/agitation of the water column but additional water was carefully added when needed, to maintain a constant depth. This methodology was to recreate waterlogged soil conditions and ensured that the roots were fully submerged, whilst the dormant seedling stem remained above the water line. At eight different time points, 10 plants were removed from the immersion tank and placed on a growing bench, adjacent to the tank. The eight time points ranged from 7 days submersion to 65 days submersion, in 7-day intervals. The number of timepoints that could be analysed was constrained by resource-availability but were spaced in response to the authors’ observation of a truffle-orchard that flooded for over 14 days without apparent deleterious impact. After removal from the submersion tank, all plants were placed on a growing bench until August–September 2016 when they were destructively sampled. All plants were sampled on the same dates, being 179–188 days after the onset of submersion, to mitigate any potential impact of them being exposed to different weather conditions. However, this meant that the post-treatment time (prior to analysis) of the treatment groups differed by as much as 58 days. The recovery periods for the 8 treatment groups were therefore 172–181, 164–173, 156–165, 148–157, 140–149, 130–139, 122–131, and 114–123 days, respectively (full details are presented in Table S1 of Online Resource 1).

### Analysis methodology

During the analysis period, all plants were destructively sampled. First, the height of each plant was measured with a measuring stick from the soil-line to the tip of the growing point. The number of attached leaves were also counted. Finally, the Rootrainer pot was opened and the root system of each plant was carefully washed to remove the majority of the growing media. The washed root system was placed against a measuring stick and the whole root was cut into three equal segments: the upper third of the root system (upper), the middle section (middle) and the bottom third (bottom). Within each root zone, 100 root tips were examined microscopically giving 300 root tips per plant and a total of 3000 for each treatment group. To prevent selection bias, once a root segment was placed under the dissecting microscope, the first root tip in the field of view was analysed and then each subsequent root tip was checked, moving along the field of view in a methodical manner, until 100 tips were counted. Each root tip was analysed for the presence/absence of *T. aestivum* EcM using the descriptions of Zambonelli et al. (1993).

## Results

Seedling survival was 100% in all categories. A simple linear regression revealed a significant relationship between leaf counts and the final height of seedlings (F(1,78) = 13.94, *p* = 0.0004) with an *R*^2^ of 0.1516. However, the total number of measured *T. aestivum* mycorrhizae per plant, from a per-plant root-tip count of 300, showed no relationship to seedling height (F(1,78) = 0.009027, *p* = 0.9246) or seedling leaf count (F(1,78) = 8.743e-007, *p* = 0.9993). Variations in mycorrhizae colonisation are therefore independent of seedling height or the number of retained leaves (data presented graphically in Fig. S1 of Online Resource 1).

**Fig. 1:**
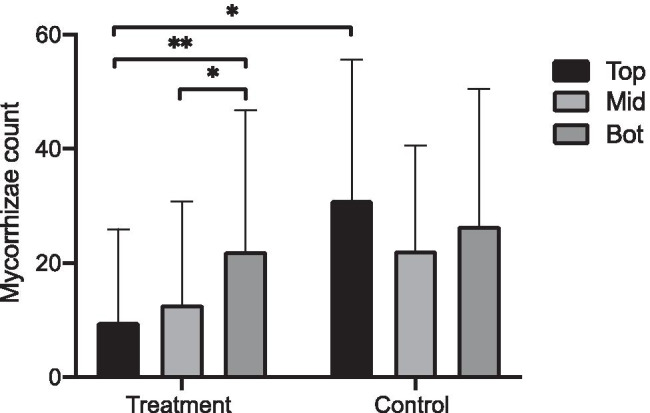
Combined *T. aestivum* mycorrhizae counts on root samples from plants in the treatment groups and the control group. Results are separated by depth of profile into the upper (Top), middle (Mid) and bottom (Bot) root zone. Error bars are standard deviation. Bars joined by brackets are significantly different at *p* < 0.05 (*) or *p* < 0.01 (**) using a post hoc Tukey’s HSD, all other comparisons between means were not significant (*p* > 0.05). Created with Prism 9 for macOS

Overall, the total number of recorded *T. aestivum* mycorrhizae per plant, from a per-plant root-tip count of 300, was significantly lower in the combined submersion treatment group (14.75) than in the control (26.5) (*t*(268) = −2.917, *p* = 0.0038). Looking at the different root horizons separately in the control and combined treatment groups, there was a statistically significant difference between mean values for profile depth between control and combined treatment groups as determined by a one-way ANOVA (*F*(5,264) = 5.168, *p* = 0.0002). Post hoc Tukey’s HSD tests revealed that the mycorrhizae count in the upper (9.64) and middle zone (12.67) were statistically significantly lower than the bottom zone (21.95) in the treatment group at the 0.05 level of significance (see Fig. 1 and Table 1).

**Table 1:** Statistical analysis results for post hoc Tukey’s HSD tests between EcM observations for the upper (Top), middle (Mid) and bottom (Bot) root zones of the treatment groups and the upper (CTop), middle (CMid) and bottom (CBot) root zones of the control group. Linear regression results for EcM observations of the upper (Top), middle (Mid) and bottom (Bot) root zones vs submersion time (Sub) and all zones combined (Combined) vs submersion time are also displayed. 95% Confidence interval (CI) represents the difference between means for multiple comparison tests and of the slope, for linear regression. Significant results with *p* < 0.05 marked with an asterisk (*)

	Mean diff	95% CI	*p* value	*R*^2^
Tukey’s multiple comparison				
Top vs. Mid	−3.025	−12.25 to 6.196	0.9353	-
Top vs. Bot	−12.31	−21.53 to − 3.091	0.0022*	-
Top vs. CTop	−21.36	−40.92 to − 1.801	0.0233*	-
Top vs. CMid	−12.46	−32.02 to 7.099	0.4489	-
Top vs. CBot	−16.76	−36.32 to 2.799	0.14	-
Mid vs. Bot	−9.288	−18.51 to − 0.06613	0.0472*	-
Mid vs. CTop	−18.34	−37.90 to 1.224	0.0804	-
Mid vs. CMid	−9.438	−29.00 to 10.12	0.736	-
Mid vs. CBot	−13.74	−33.30 to 5.824	0.336	-
Bot vs. CTop	−9.05	−28.61 to 10.51	0.7692	-
Bot vs. CMid	−0.15	−19.71 to 19.41	> 0.9999	-
Bot vs. CBot	−4.45	−24.01 to 15.11	0.9867	-
CTop vs. CMid	8.9	−17.18 to 34.98	0.9241	-
CTop vs. CBot	4.6	−21.48 to 30.68	0.9959	-
CMid vs. CBot	−4.3	−30.38 to 21.78	0.997	-
Linear regression				
Combined vs. Sub	-	−0.2600 to 0.01281	0.0755	0.01321
Top vs. Sub	-	−0.1780 to 0.2030	0.8962	0.00022
Mid vs. Sub	-	−0.2951 to 0.1265	0.4286	0.00805
Bot vs. Sub	-	−0.5806 to − 0.01756	0.0376*	0.05424

The upper root zone mycorrhizae count of the treatment group was also statistically significantly lower than the upper root zone in the control group (31.00) but all other comparisons were not significant. The impact of submersion is therefore displayed significantly in the upper and mid layers of the root zone.

Although the mycorrhizae counts were lower in the upper root zones of the treatment groups root system, the duration of flood event did not appear to be as influential as expected. The total mycorrhizae counts for seedlings submerged at the first time point (day 7) vs the control were significantly different (*t*(58) = −2.1864, *p* = 0. 0164), but increased duration of flood event did not seem to impact this further. A simple linear regression revealed no significant relationship between duration of submersion from 7 to 65 days and total seedling mycorrhizae counts (F(1, 238) = 3.186, *p* = 0.0755) with an *R*^2^ of 0.01321. However, if the root zones are analysed independently, then for the bottom layer only, there is a significant relationship between duration of submersion from 7 to 65 days and mycorrhizae counts (F(1, 78) = 4.473, *p* = 0.0376) with an *R*^2^ of 0.05424 (see Table 1 and Fig. S2 of Online Resource 1). The shortest submersion period in this study (7 days) is therefore enough time to significantly impact mycorrhizae, but beyond this time point, there is only a relationship between submersion time and mycorrhizae counts in the lowest root zone.

## Discussion

Full submersion for 7 days is enough to cause a significant impact on EcM numbers. However, beyond this time-point, the duration of submersion appears to be less important than expected. Extending the duration of submersion, up to a full 65 days, does not further add to this deleterious impact and the survival of EcM for this duration is unexpected. The division of root systems into three distinct root zones allows some further exploration of the results. There does not appear to be any difference between the lower and middle root zones in terms of mycorrhizae numbers between the treatment and control groups, but in the upper root zone, there is. Stratification of temperature, methane and dissolved oxygen occurs in even shallow bodies of water (Ford et al. 2002). EcM requires oxygen for respiration, and within saturated soil, oxygen gradients also exist with the diffusion of oxygen being further constrained by soil texture and porosity (Neira et al. 2015); the changes to physiochemical properties in such conditions may also be significant (Vepraskas et al. 2001). As a whole, these changes may in part explain the observed reductions in *T. aestivum* EcM, to flooding. If the expectation of greater oxygen diffusion at the surface layer and progressively more hypoxic conditions with depth is correct, the upper zones would likely be less responsive to flooding. Instead, we see the inverse, and this may be explained by recovery methods.

The post-submersion observation of EcM may represent resilience, recovery through new mycorrhizae development or, most likely, a combination of both. Due to the elapsed time between the end of treatment within each group and the analysis date, there is potential for a mycorrhizal recovery method (or methods) occurring prior to analysis.

**Fig. 2:**
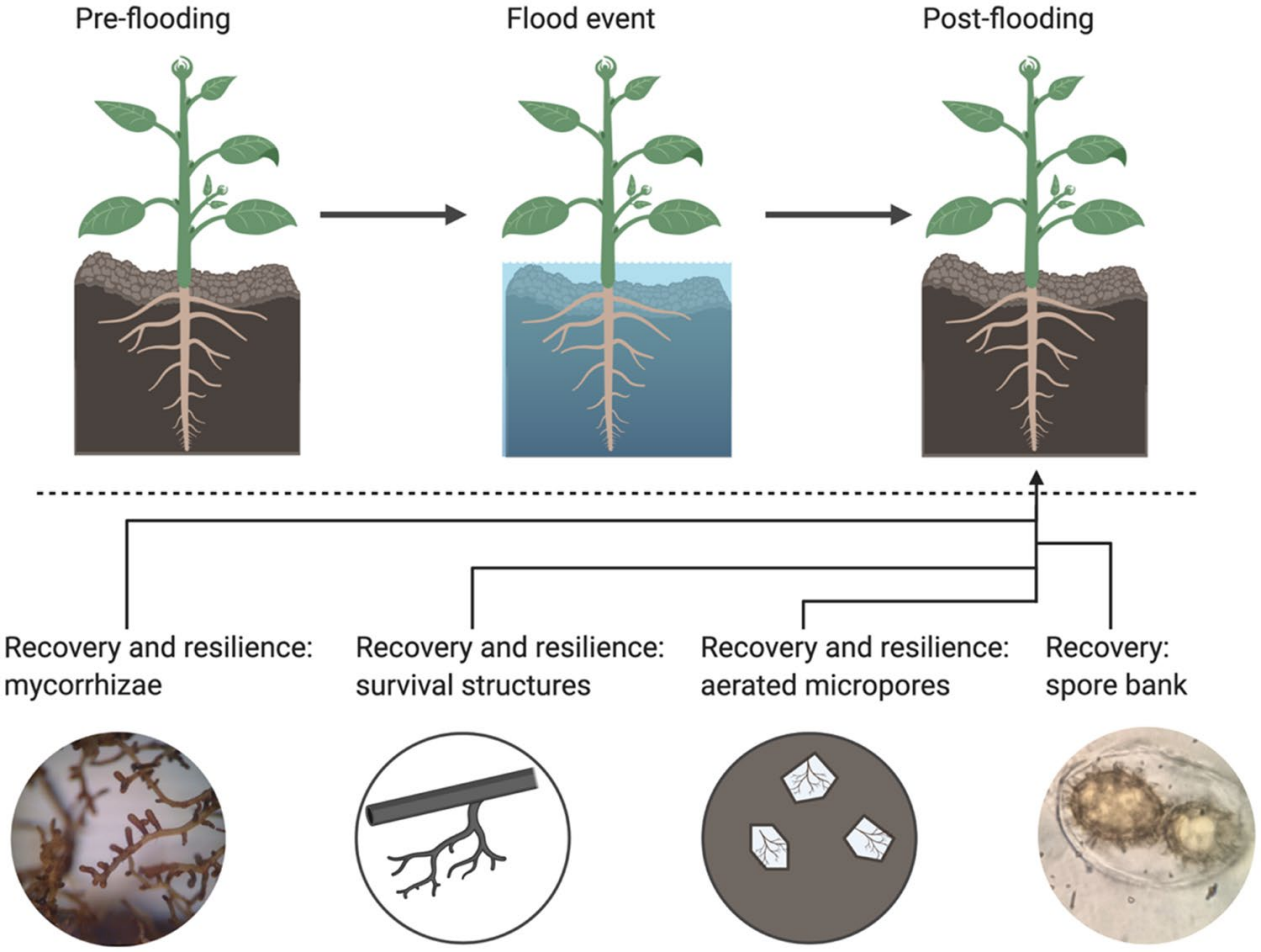
Diagrammatic representation of possible EcM recovery and resilience methods to flood events. These include the development of new mycorrhizae from: existing mycorrhizae, survival structures as aggregated hyphae, mycelial survival in aerated micro-pockets and the existing spore bank. Created with Biorender.com

*T. aestivum* belongs to the Ascomycota phylum, a group in which spores are formed within an ascus and this may afford the spores some mechanical protection. Mycorrhizae development from spores is well understood and some species within the *Rhizopogon* genus have shown increased ability to infect tree roots, with duration in the spore bank: up to a current maximum tested age of 4 years (Bruns et al. 2009). Further, species within the *Tuber* genus can survive significant periods of storage in inhospitable environments, such as drying for 3 months (Bonito et al. 2012) or transit through mammalian digestive tracts (Piattoni et al. 2014). Persistence of spores from the initial inoculation of seedlings within this experiment, therefore, represents one possible method by which new roots may become infected post-submersion. *T. aestivum* may also form clusters of aggregated hyphae, including as fruiting body attachment structures (Deveau et al. 2019), and these can act as survival refugia with nutritional stores from which mycelial expansion may occur. It is also a possibility that mycelial fragments may also survive within micro-pockets of aeration within the soil matrix (see Fig. 2).

Development of new EcM from such refugia is likely aided by carbon transfer from the plant partner. During periods of host plant photosynthetic dormancy, carbon may arise from stored reserve compounds and hydrolyzed during cool-weather events (Le Tacon et al. 2013). Spatial differences in refugia, such as a greater residual spore load in the lower zone, mechanically transported by repeated watering post-inoculation may explain why EcM levels are higher in the lowest zone post-submersion. Further investigation is needed to identify if such differences exist.

Another factor that needs to be considered is that the treatment groups also differ in their recovery time, a term here applied to the duration between the end of the submersion period and the analysis date. The group with the shortest submersion duration (7 days) had a greater potential recovery time of 58 days compared with the group subjected to the greatest submersion time. However, it is possible that increased recovery time may not have a positive impact on mycorrhizae development. For example, *T. aestivum* favours alkaline conditions (Thomas 2012) and the soil pH modifier used within this experiment is water soluble (CaCO_3_) and especially so in rainwater which has a higher concentration of dissolved CO_2_. Therefore, if submersion causes a change to sub-optimal pH levels, a longer ‘recovery time’ in such conditions may be deleterious. Unfortunately, changes in substrate physio-chemical properties post-submersion were not analysed in this study. Further, experimentation is needed to assess if these differences in recovery time are significant and what changes to the soil physiochemical properties have occurred.

## Conclusion

The results of this study support, and in a dose-dependent manner, the previously suggested impacts that flooding can have on EcM. AM tend to dominate in soils frequently subject to saturation, but here, it is shown that even though the relative quantity of EcM on a host plant is significantly damaged with short durations of flooding, they also display a surprising degree of resilience to inhospitable conditions. So much so, that even after 65 days of flooding, there is persistence of EcM, most likely from a mix of resilience and recovery methods. Further, the surprisingly higher levels of EcM, post-flooding, in the lower soil zones may be indicative of the importance of recovery mechanisms.

These results provide some insight into the tolerances of EcM to saturated soils. The results are not only of benefit to mycologists but also have implications for ecologists working on a wide range of plant community and soil-plant interaction topics. Finally, the results also have a practical element for management in timber and nut production using trees that form EcM and especially for truffle-cultivators, where climate change presents an expectation for extreme rainfall events and flooding to increase in severity and frequency.

## Supplementary information

Below is the link to the electronic supplementary material.

Supplementary file1 (PDF 330 KB)

## Data Availability

Full datasets available on request.
