# Supplementary material for: Ectomycorrhiza resilience and recovery to extreme flood events in Tuber aestivum and Quercus robur
Source: Mycorrhiza. 2021 May 13;31(4):511–7. doi: 10.1007/s00572-021-01035-4 (PMC8266694; doi:10.1007/s00572-021-01035-4)
Supplement: Supplementary file 1 — Supplementary file1 (PDF 330 KB) [file 572_2021_1035_MOESM1_ESM.pdf]

Electronic supplementary material

Ectomycorrhiza resilience and recovery to extreme flood events in *Tuber aestivum* and *Quercus robur*

Author; P. W. Thomas[1,2,*]

Journal; *Mycorrhiza*

[1] *University of Stirling, Faculty of Natural Sciences, Stirling, FK9 4LA, UK.*

[2] *Mycorrhizal Systems Ltd, Lancashire, PR25 2SD, UK.*

[*] *Corresponding author: paul.thomas@stir.ac.uk, +44(0) 333 2427831.*

Table S1. Details for the experimental groups used in this study. Dates are calculated from the start of the year, with the 1st of January 2016 being day 1 and, for example, the 25th of January 2016, being day 25. Note: all treatment groups were subject to analysis on the same dates and this resulted in group S1 having a greater recovery time from group S8 by 58 days.

Group	Submersion day	Days submerged	Analysis day	Recovery (days)
S1	25	7	204-213	172-181
S2	25	15	204-213	164-173
S3	25	23	204-213	156-165
S4	25	31	204-213	148-157
S5	25	39	204-213	140-149
S6	25	49	204-213	130-139
S7	25	57	204-213	122-131
S8	25	65	204-213	114-123

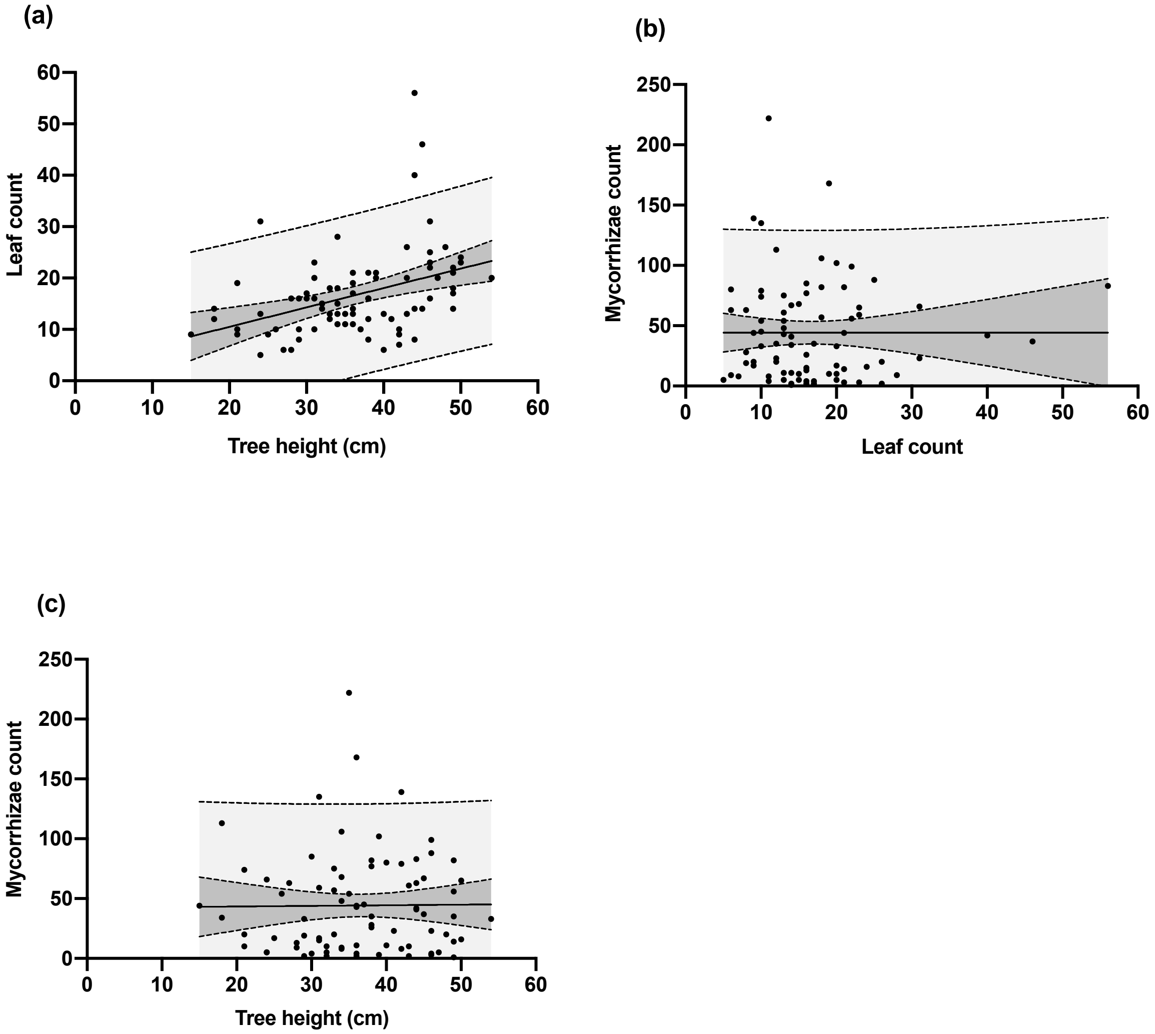

Fig S1 Regression analysis of whole data showing leaf count plotted against tree height (a), total mycorrhizae count plotted against leaf count (b) and total mycorrhizae count plotted against tree height (c). Confidence level or range (95%) and predictive intervals are also displayed. Created with Prism 9 for macOS

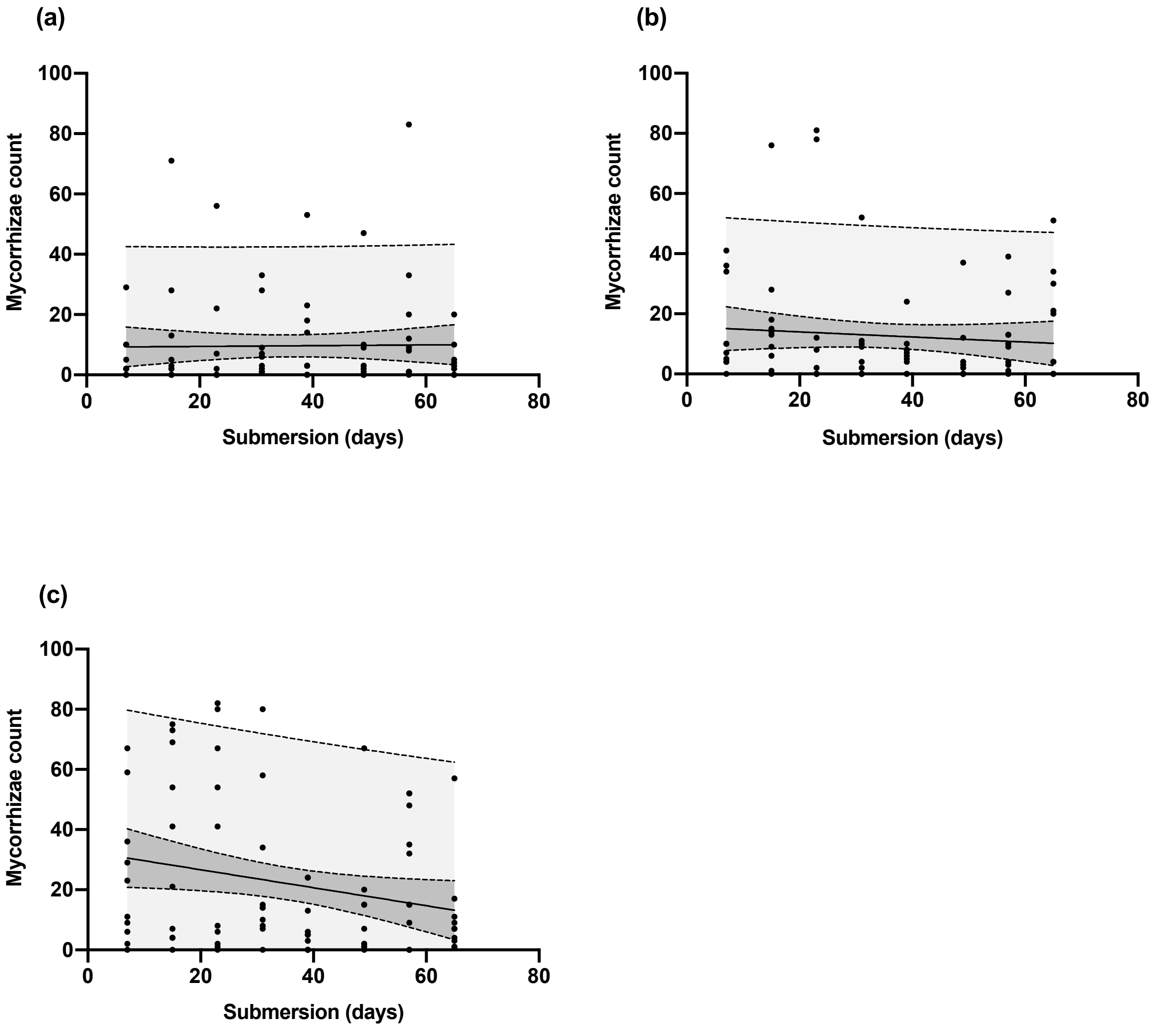

Fig S2 Regression analysis of mycorrhizae counts for the top (a), middle (b) and bottom (c) root zones of treatment group plants, plotted against submersion time (days). Confidence level or range (95%) and predictive intervals are also displayed. Created with Prism 9 for macOS
